# Period-3 dominant phase synchronisation of *Zelkova serrata*: border-collision bifurcation observed in a plant population

**DOI:** 10.1038/s41598-019-50815-8

**Published:** 2019-10-30

**Authors:** Kenshi Sakai, Yoshinobu Hoshino, Awadhesh Prasad, Atsuko Sugawara Fukamachi, Akira Ishibashi

**Affiliations:** 1 Division of Environmental and Agricultural Engineering, Institute of Agriculture, Tokyo University of Agriculture and Technology, Tokyo, 183-8509 Japan; 2 Division of Environment Conservation, Institute of Agriculture, Tokyo University of Agriculture and Technology, Tokyo, 183-8509 Japan; 3 Department of Physics and Astrophysics, University of Delhi, Delhi, 110007 India; 4 Department of Environment Conservation, Graduate School of Agriculture, Tokyo University of Agriculture and Technology, Tokyo, 183-8509 Japan

**Keywords:** Computational models, Nonlinear phenomena

## Abstract

The population synchrony of tree seed production has attracted widespread attention in agriculture, forestry and ecosystem management. Oaks usually show synchronisation of irregular or intermittent sequences of acorn production, which is termed ‘masting’. Tree crops such as citrus and pistachio show a clear two-year cycle (period-2) termed ‘alternate bearing’. We identified period-3 dominant phase synchronisation in a population of *Zelkova serrata*. As ‘period-3’ is known to provide evidence to imply chaos in nonlinear science, the observed period-3 phase synchronisation of *Zelkova serrata* is an attractive real-world phenomenon that warrants investigation in terms of nonlinear dynamics. Using the Hilbert transform, we proposed a procedure to determine the fractions of periods underlying the survey data and distinguished the on-year (high yield year) and the off-year (low yield year) of the masting. We quantified the effects of pollen coupling, common environmental noise and individual variability on the phase synchronisation and demonstrated how the period-3 synchronisation emerges through a border-collision bifurcation process. In this paper, we propose a model that can describe diverse behaviours of seed production observed in many different tree species by changing its parameters.

## Introduction

The highly synchronised fluctuation of annual seed production is common in perennial plant species. Among such species, acorn masting has particularly attracted interest in many fields of research^1–4^. In silviculture, prediction of masting behaviour in forest stands is necessary for approaches that promote natural regeneration^5,6^. As acorns are a substantial food source for wild animals^2^, it is important to understand masting for ecological management^7–10^. ‘Alternate bearing’ refers to tree crops that produce heavy crops one year (the ‘on’ year) and light crops the following year (the ‘off’ year). Citrus (e.g., oranges, lemons and mandarins), pistachios and chestnuts are crops that show pronounced alternate bearing^11–15^. Acorn masting and alternate bearing also have been investigated in terms of the synchronisation of ensembles of trees^16–19^. In nonlinear physics, the synchronisation of ensembles of oscillators is known to be caused by mutual coupling or common identical noise^20,21^. Many types of coupling, such as indirect global and local coupling^16–19^ and direct coupling^22^, have been investigated. The common noise-induced synchrony^23–27^ is known as the Moran effect in population ecology. In a 15-year field survey, we observed period-3 dominant phase synchronisation in a population of 106 individuals of *Zelkova serrata*. To date, the majority of masting behaviour has been recognised as irregular and/or intermittent sequences and alternate bearing of tree crops is generally period-2. Therefore, the period-3 dominant synchronisation identified here is unique. In particular, period-3 is a special term in nonlinear dynamics, as it has been proven that certain dynamics of period-3 can generate any periods including chaos^28^. Thus, elucidation of the mechanism of period-3 phase synchronisation in *Zelkova serrata* will contribute to understanding the variety of periods synchrony reported in many perennial plant species.

The objective of this study was to clarify the mechanism underlying the period-3 dominant synchronisation in the population of *Zelkova serrata* surveyed. We developed a method to quantify various periodic compositions coexisting in an ensemble time series. We demonstrated a globally coupled map of the resource budget model (hereafter GCM-RBM)^16^ to model the period-3 dominant phase synchronisation of *Zelkova serrata* as its masting behaviour.

The GCM-RBM has been commonly used to model the population synchrony of cross-pollinated plants^16–18^. At a certain magnitude of coupling strengths (*β*), the group consisting of *N* RBMs is strongly synchronised so that its dynamics can be represented by a one-dimensional map. We demonstrate how the period-3 emerges from a tangential bifurcation of the GCM-RBM whose map (characterised as a border-collision bifurcation) is piecewise smooth and piecewise monotonic^29,30^. We also estimated control parameters (*R*_C_ and *β*) of the GCM-RBM for the survey data for *Zelkova serrata*. The proposed approach is expected to be a powerful method to understand the mechanisms of synchronisation in various perennial plant species.

## Materials and Methods

### Field survey

*Zelkova serrata* is a diclinous monoecious tree distributed in East Asia and flowers in April and May. The leaf colour changes to vivid red in autumn and the leaves on fruiting twigs change colour much earlier than those on non-fruiting twigs (see Supplementary Fig. S1). On the basis of visual inspection from the ground in two weeks of mid-November, the seed production level was classified into 10 classes. This method is popular in vegetation surveys^31,32^. Data from a population of 106 trees acquired over 15 years from 2003 to 2017 were analysed. This primary survey was conducted in the area bounded by longitude 139°28′45.57″E to 139°28′45.84″E and latitude 35°40′11.99″N to 35°40′31.90″N in Fuchu City, Tokyo, Japan. We also conducted a additional survey for 48 trees since 2006 in the area bounded by longitude 139°41′27.64″E to 139°41′40.58″E and latitude 35°41′25.04″N to 35°41′27.30″N in Shinjuku District located 20 km west of Fuchu City.

#### Fraction of the period-*Q* sequence

For identification of the ‘on-year’ and the ‘off-year’ for a single time series *x*(*t*), we defined the flag index *ON*(*t*) as a step function by employing phase-based and amplitude-based definitions.

As the phase angle *θ*(*t*) of a single time series *x*(*t*) is needed to define *ON*(*t*), it is given by

1 $$\theta (t)={\rm{angle}}({\rm{HT}}[X(t)-\bar{X}])$$

where HT is the Hilbert transform of the true signal, $\bar{X}$ is the time average of *X*(*t*)^31–38^.

We used MATLAB^®^ for this calculation with the signal processing toolbox code ‘hilbert’^32,33^ for {*X*(*t*, *i*); *t* = 1, …, *T*, *i* = 1, …, *N*} using

*θ*(:, :) = angle(hilbert(*X*(:, :) − mean(*X*(:, :), 1))).

In the phase-based definition, we used the phase $\frac{\pi }{2}$ as a threshold to distinguish the ‘on-year’ and the ‘off-year’. $|\theta (t)| < \frac{\pi }{2}$ and $|\theta (t)|\ge \frac{\pi }{2}$ correspond to the on-year and the off-year, respectively.

2 $$O{N}_{P}(t)=\{\begin{array}{c}1\,,\,\,\,\,|\theta (t)| < \frac{\pi }{2}\\ 0\,,\,\,\,\,|\theta (t)|\ge \frac{\pi }{2}\end{array}$$

In the amplitude-based definition, we used the time average of *x*(*t*) as a threshold to distinguish the ‘on-year’ and the ‘off-year’. When *x*(*t*) is larger than $\bar{x}=\mathop{\sum }\limits_{t=1}^{T}x(t)$, then year *t* is considered to be the ‘on-year’.

3 $$O{N}_{A}(t)=\{\begin{array}{c}1\,,\,\,\,\,x(t) > \bar{x}\\ 0\,,\,\,\,\,x(t)\le \bar{x}\end{array}$$

Matching between *ON*_*A*_(*t*) and *ON*_*P*_(*t*) was examined with 1600 combinations of the two control parameters (*β* and *R*_C_) used in Fig. 7 to show that the two indices were identical.

**Figure 1:**
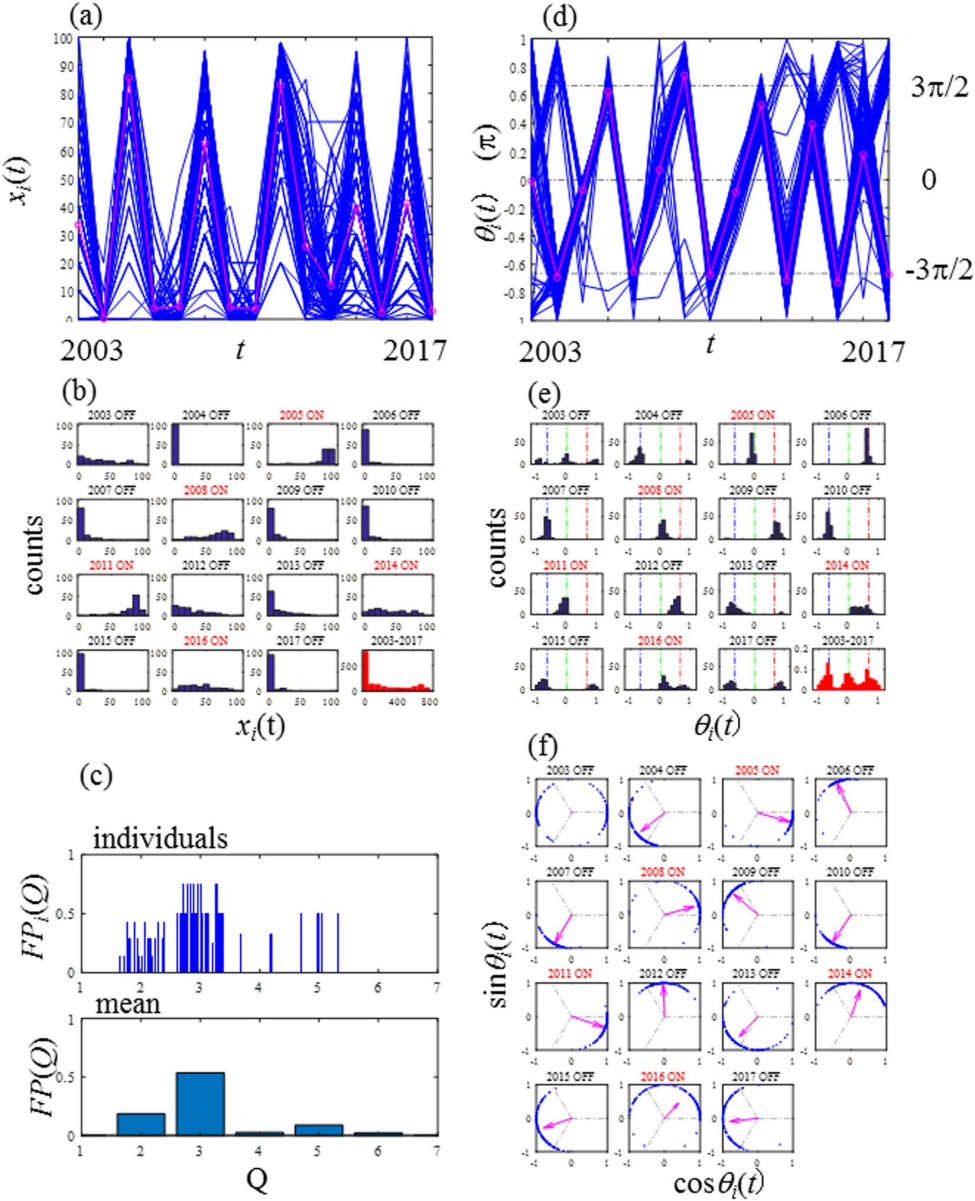
Seed production of 106 *Zelkova serrata* trees over 15 years (2003–2016) at Fuchu City, Tokyo, Japan. (**a**) Seed production *x*_*i*_(*t*). The mean seed production *X*(*t*) (thick solid line in magenta) demonstrates a clear period-3 pattern. (**b**) Histograms of *x*_*i*_(*t*). (**c**) Fractions of period-*Q* for individuals, *FP*_*i*_(Q), and for the population, *FP*(*Q*). (**d**) Phase of the seed production *θ*_*i*_(*t*) with the median of phase *Θ*(*t*) in magenta. (**e**) Histograms of *θ*_*i*_(*t*). (**f**) Circle maps.

**Figure 2:**
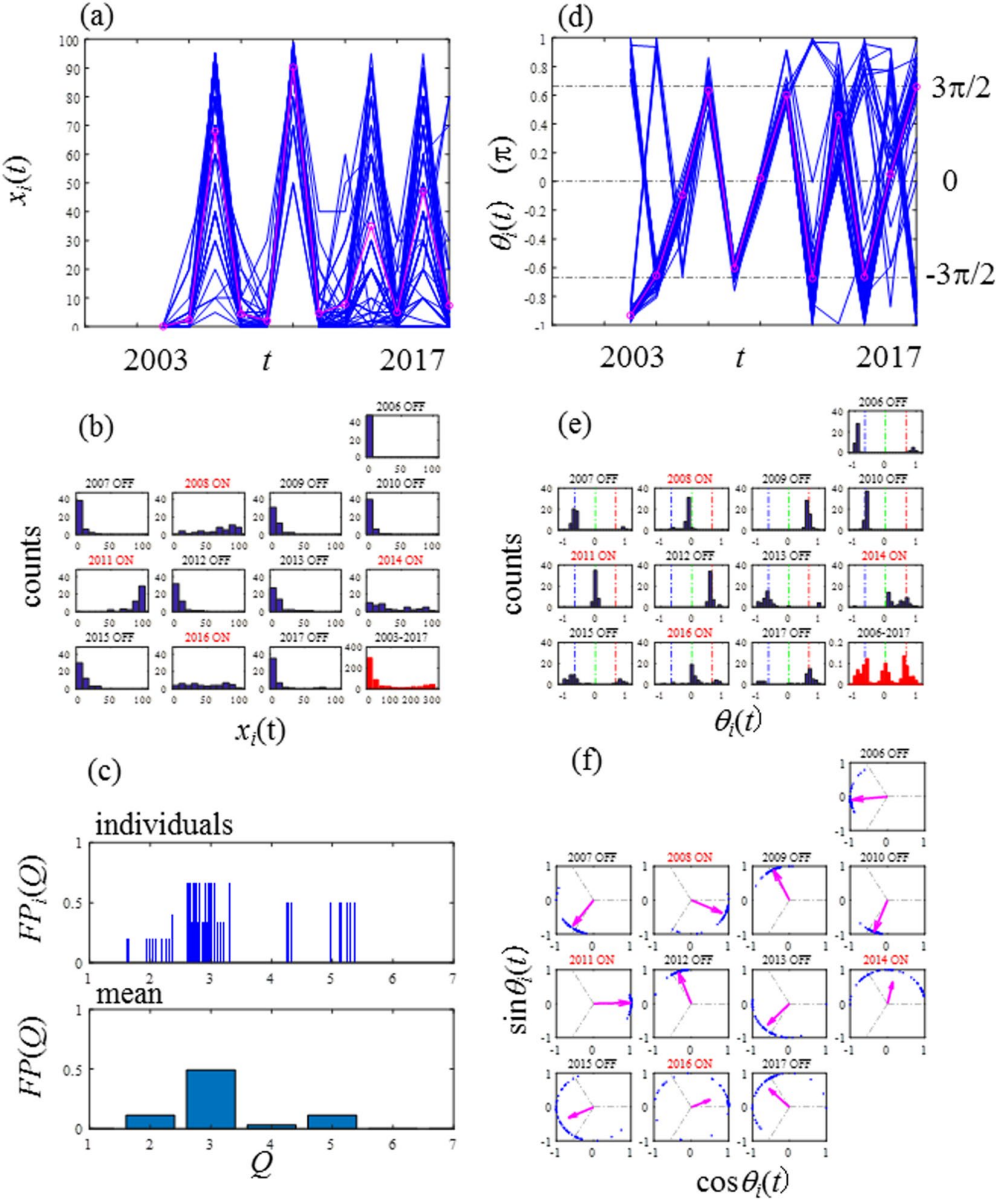
Seed production of 48 *Zelkova serrata* trees over 12 years (2006–2017) at Shinjuku District, Tokyo, Japan. (**a**) Seed production *x*_*i*_(*t*). The average production *X*(*t*) (thick solid line in magenta) demonstrates a clear period-3 pattern. (**b**) Histograms of *x*_i_(*t*). (**c**) Fractions of period-Q for individuals; *FP*_*i*_(*Q*) and the population; *FP*(*Q*). (**d**) Phase of the seed production *θ*_*i*_(*t*) with the median of phase *Θ*(*t*) in magenta. (**e**) Histograms of *θ*_*i*_(*t*). (**f**) Circle maps.

**Figure 3:**
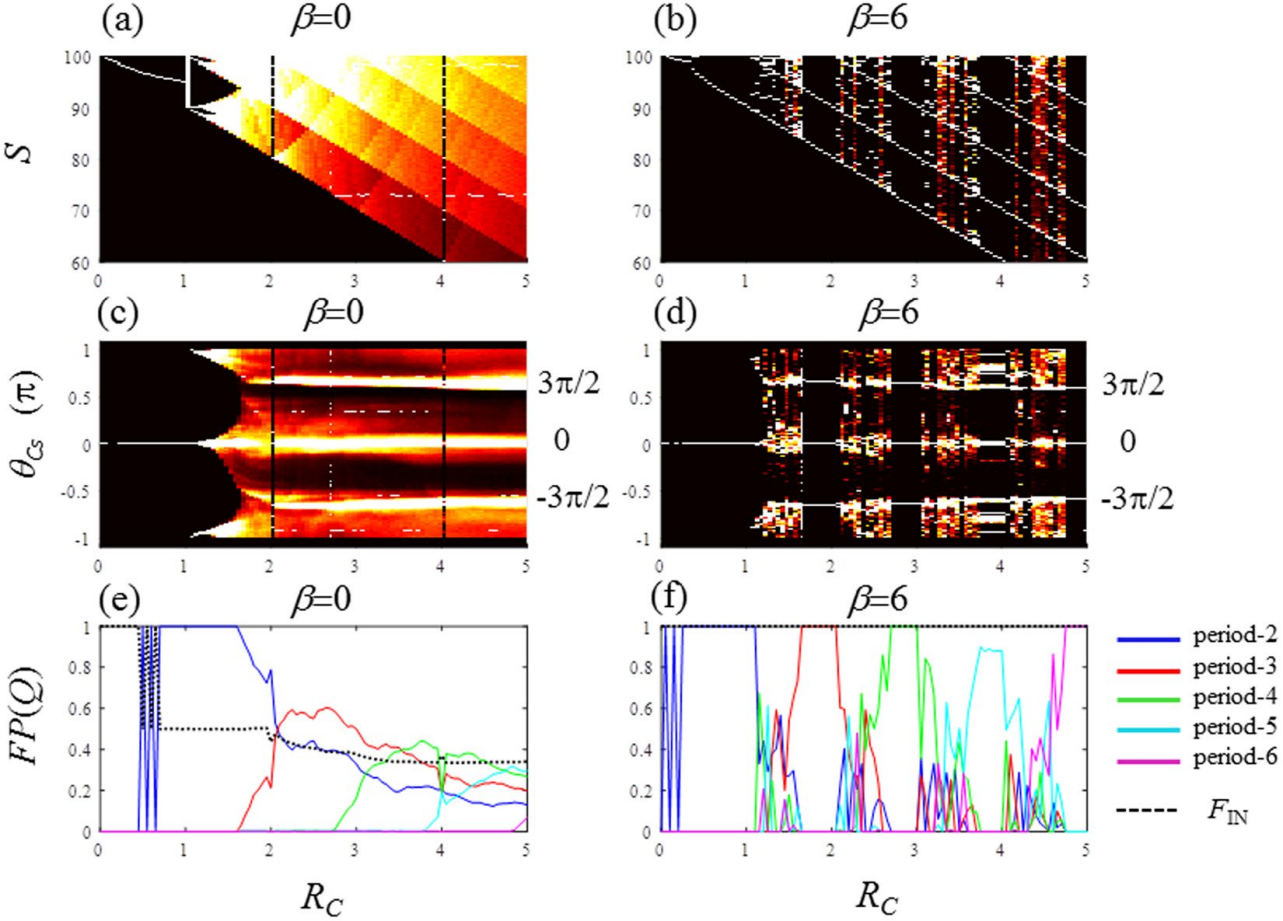
Period-adding bifurcation of the globally coupled map of the resource budget model for the noise-free condition (*e*_I_ = 0). (**a**–**d**) Density bifurcation diagrams for *S*, *C*_*S*_ and *θ*_*Cs*_: (**a**) S: *β* = 0, (**b**) S: *β* = 6, (**c**) *θ*_*Cs*_: *β* = 0, and (**d**) *θ*_*Cs*_: *β* = 6. (**e,f**) Fraction of periods *FP*(*Q*) for (**e**) *β* = 0, and (**f**) *β* = 6. *FP*(2): blue, *FP*(3): red, *FP*(4): green, *FP*(5): cyan, and *FP*(6): magenta, *F*_IN_: dotted line.

**Figure 4:**
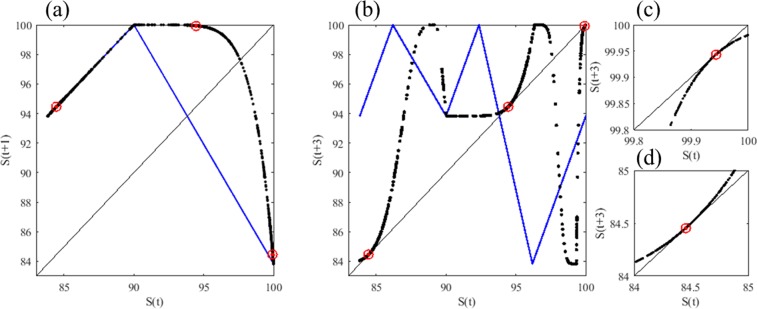
Mechanism generating the period-3 cycle in the tangent bifurcation of the globally coupled map of the resource budget model (GCM-RBM) for *β* = 0 (blue thin line) and *β* = 6 (black dots). The GCM-RBM is a piecewise smooth and piecewise monotonic map and has border collision period-doubling bifurcations. (**a**) First iterated plots of *S*, (**b**) third iterated plots of *S*, (**c**) magnified plots around *S* = 99.9438 and (**d**) magnified plots around *S* = 84.4523.

**Figure 5:**
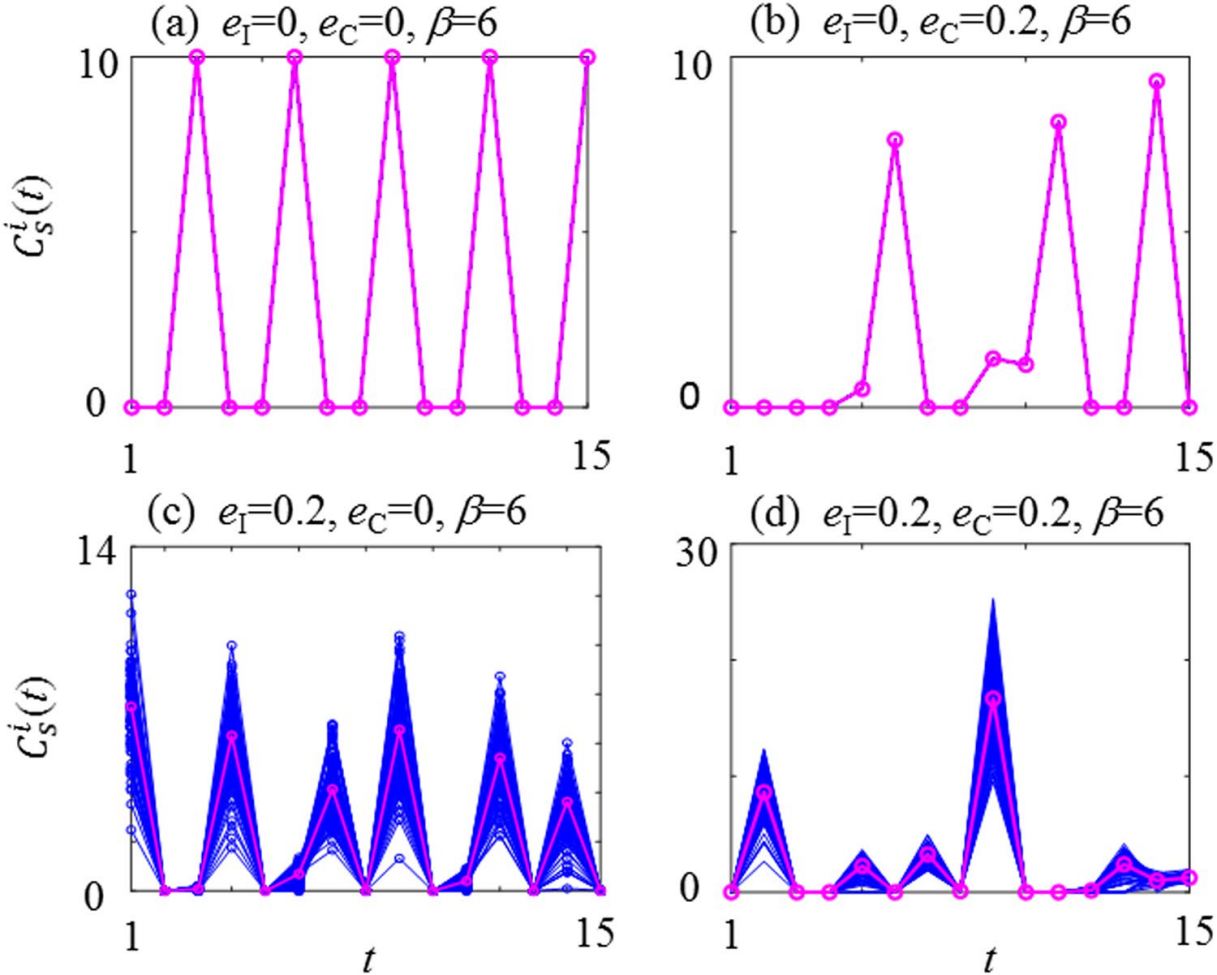
Effects of common noise (*e*_C_) and individual noise (*e*_I_) on seed production (*C*_S_) of *Zelkova serrata*. (**a**) noise free: *e*_I_ = 0, *e*_C_ = 0; (**b**) common noise only: *e*_I_ = 0 , *e*_C_ = 0.2; (**c**) individual noise only: *e*_I_ = 0.2, *e*_C_ = 0.; (**d**) common noise and individual noise: *e*_I_ = 0.2, *e*_C_ = 0.2, at *R*_*C*_ = 2 and *β* = 6 for (**a**-**d**). The average seed productions are plotted in magenta.

**Figure 6:**
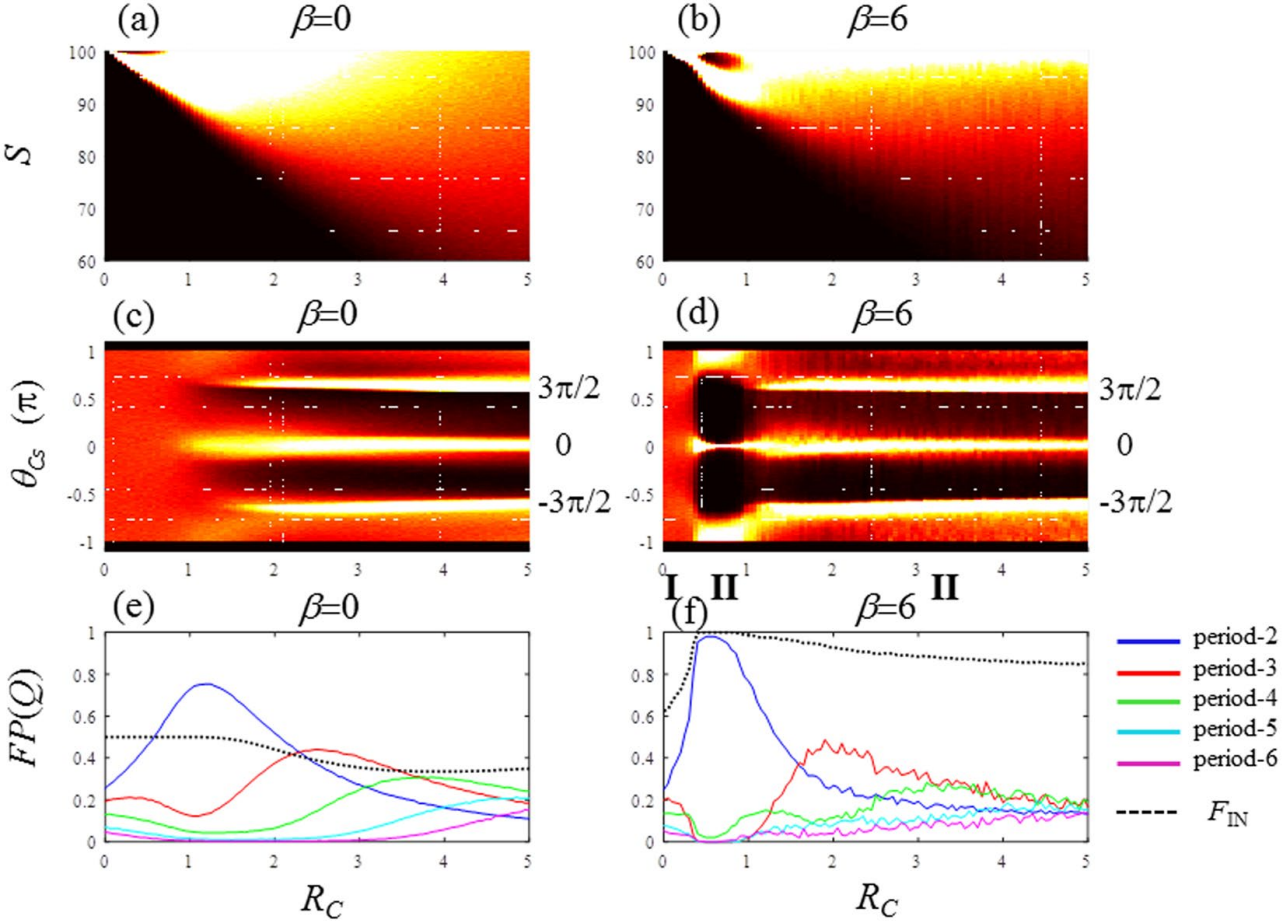
Period-adding bifurcation of the globally coupled map of the resource budget model for the noise-imposed condition (*e*_I_ = 0.2). (**a**–**d**) Density bifurcation diagrams for *S*, and *θ*_Cs_: (**a**) *S*: *β* = 0, **(b**) *S*: *β* = 6, (**c**) *θ*_Cs_: *β* = 0, and (**d**) *θ*_Cs_: *β* = 6. (**e,f**) Fraction of periods *FP*(Q) for: (**e**) *β* = 0, and (**f**) *β* = 6. *FP*(2): blue, *FP*(3): red, *FP*(4): green, *FP*(5): cyan and *FP*(6): magenta, *F*_IN_: dotted line.

**Figure 7:**
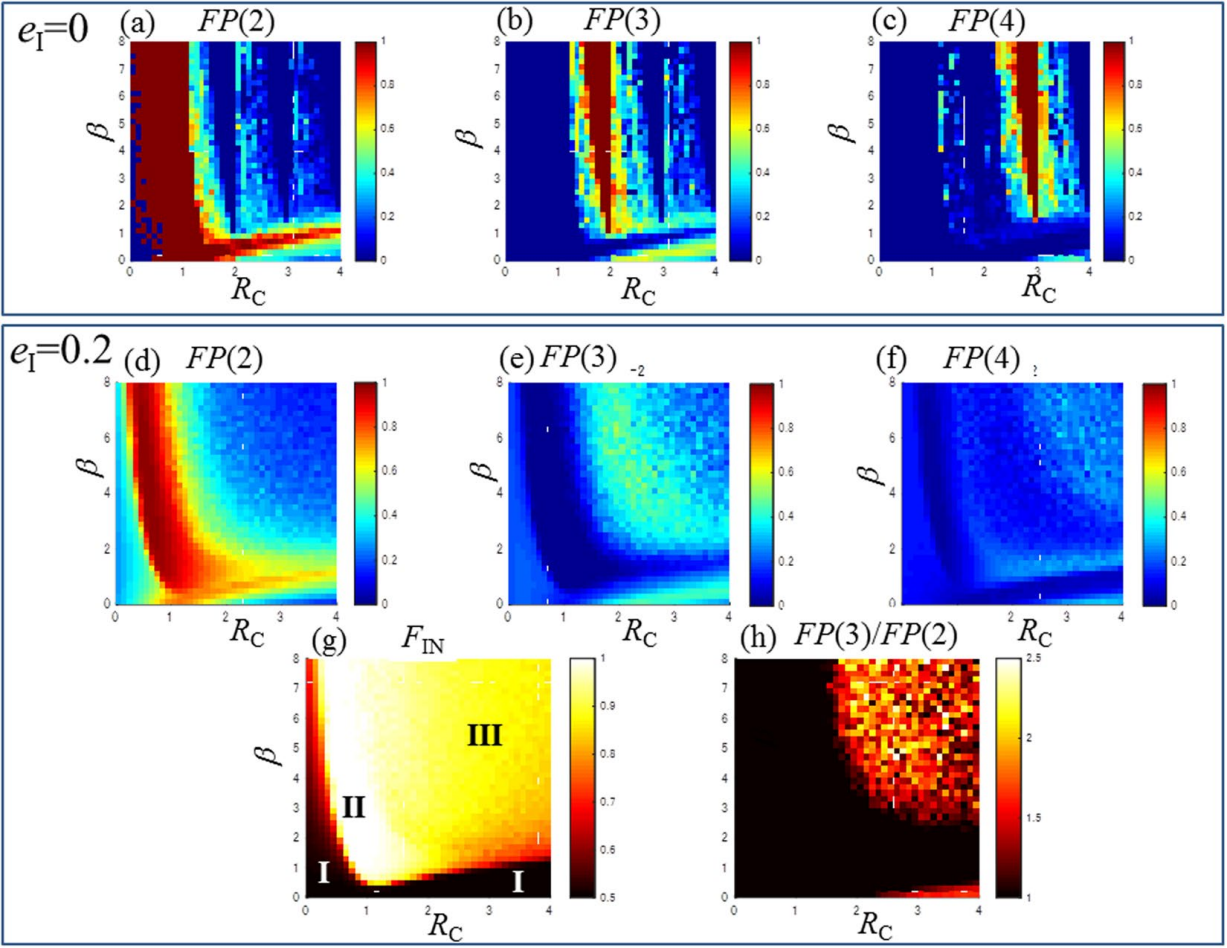
Estimation of the control parameters (*R*_C_ and *β*) of the globally coupled map of the resource budget model for *Zelkova serrata*. (**a**) Fraction of period-2 *FP*(2) for *e*_I_ = 0, (**b**) fraction of period-3 *FP(*3) for *e*_I_ = 0, (**c**) fraction of period-4 *FP*(4) for *e*_I_ = 0, (**d**) fraction of period-2 *FP*(2) for *e*_I_ = 0.2, (**e**) fraction of period-3 *FP*(3) for *e*_I_ = 0.2, (**f**) fraction of period-4 *FP*(4) for *e*_I_ = 0.2, (**g**) fraction of in-phase *F*_IN_ for *e*_I_ = 0.2 and (**h**) *FP*(3)/*FP*(2) ratio for *e*_I_ = 0.2.

The afore-mentioned two definitions of *ON*(*t*) can be expanded to the ensemble {*x*_i_(*t*); *t* = 1, 2, …, *T*, i = 1, 2, …, *N*} to obtain *ON*_*i*_(*t*) for each tree *i* as follows,

4 $$O{N}_{A,i}(t)=\{\begin{array}{c}1\,,\,\,\,\,{x}_{i}(t) > {\bar{x}}_{i}\\ 0\,,\,\,\,\,{x}_{i}(t)\le {\bar{x}}_{i}\end{array},$$

where

$${\bar{x}}_{i}=\frac{1}{T}\mathop{\sum }\limits_{t=1}^{T}{x}_{i}(t),$$

and

5 $$\,O{N}_{P,i}(t)=\{\begin{array}{c}1\,,\,\,\,\,|{\theta }_{i}(t)| < \frac{\pi }{2}\\ 0\,,\,\,\,\,|{\theta }_{i}(t)|\ge \frac{\pi }{2}\end{array}.$$

To investigate the phase synchronisations of masting in a population of trees, it is important to observe the composition of periods in individual trees and the population. Here, we propose a practical means to determine the composition of periodic components. For instance, period-2 and period-3 sequences are defined as ‘ON ⇒ OFF ⇒ ON’ and ‘ON ⇒ OFF ⇒ OFF ⇒ ON’, respectively. Thus, a period-*Q* sequence is defined as the sequence where one ‘on-year’ at year *t* is followed by *Q* − 1 for ‘off-year’ and ‘on-year’ arises at year *t* + *Q*. The fraction of period-*Q* in the *i*^th^ tree’s time series is determined by

6 $$F{P}_{i}(Q)=\frac{Q}{(T-1)-\,{\rm{mod}}(T-1,Q)}\mathop{\sum }\limits_{t=1}^{T-Q}[O{N}_{i}(t)O{N}_{i}(t+Q)\mathop{\prod }\limits_{j=2}^{Q-1}(1-O{N}_{i}(t+j))].$$

where *ON*_i_(*t*) is determined by Eqs (4, 5).

The fraction of period-*Q* representing a population is given by

7 $$FP(Q)={\rm{mean}}(F{P}_{i}(Q)),\,i=1,2,\ldots ,N.$$

The median and mode can be used to represent a population as well as the mean.

#### Measures of synchrony

Given that we focused on the phase synchronisation, we employed the notion of in-phase and out-of-phase analysis^22^. The fraction of in-phase in a population {*x*_i_(*t*); *i* = 1, 2, …, *N*, *t* = 1, 2…, *T*} is also used^22^ to measure the phase synchronisation of a population of trees. If the arbitrary pair of *x*_*i*_(*t*) and *x*_*j*_(*t*) show in-phase behaviour between year *t* and year *t* + 1, then

Let *x*_*i*_(*t*) be the yield of the *i*^th^ tree in year *t*, and define *ϕ* (*i*, *j*, *t*) as the phase between the *i*^th^ and *j*^th^ tree:

8 $$\varnothing (i,j,t)=\{{x}_{i}(t+1)-{x}_{i}(t)\}\times \{{x}_{j}(t+1)-{x}_{j}(t)\}.$$

The fractions of in-phase movements of the *i*^th^ tree relative to the remaining trees in the population is defined as

9 $${f}_{{\rm{IN}}}^{i}(t)=\frac{1}{N-1}\mathop{\sum }\limits_{j=1,j\ne i}^{N}{{\rm{H}}}_{{\rm{IN}}}[\varnothing (i,j,t)].$$

where H_IN_ is given as Heaviside step function, however, H_IN_ = 1, when *x*_*i*_(*t* + 1) − *x*_*i*_(*t*) = *x*_*j*_(*t* + 1) − *x*_*j*_(*t*) = 0.

The fraction of in-phase of the population (size *N*) at time *t* is expressed by *f*_IN_(*t*).

*F*_IN_ denotes the time average of *f*_IN_(*t*).

### Model

The resource budget model (RBM) is described below and has been used previously to model masting and alternate bearing^16,39^. Let *S*_*i*_(*t*) be the amount of resource reserves at the beginning of year *t* for tree *i*. If the accumulated resource ${S}_{i}(t)+{P}_{S}$ exceeds the threshold of the pool (*L*_T_), then the excess amount *S*_*i*_(*t*) + *P*_*S*_ − *L*_*T*_ is used for flowering ${C}_{f}^{i}(t)$.

10 $${S}^{i}(t+1)=\begin{array}{c}{S}^{i}(t)+{P}_{S},\,\,\,\,\,\,\,\,\,\,\,\,\,\,\,\,\,\,\,\,\,\,\,\,\,\,\,\,\,\,\,\,\,\,\,\,{S}^{i}(t)+{P}_{S}\le {L}_{T}\\ {S}^{i}(t)+{P}_{S}-{C}_{f}^{i}(t)-{C}_{a}^{i},\,\,\,{S}^{i}(t)+{P}_{S} > {L}_{T}\end{array}$$

11 $${C}_{f}^{i}(t)={S}^{i}(t)+{P}_{S}-{L}_{T}$$

The cost of pollinating flowers and bearing fruit is designated by ${C}_{a}^{i}(t)$. The cost ratio *R*_*C*_ = ${C}_{a}^{i}/{C}_{f}^{i}$ is a constant.

12 $${C}_{a}^{i}(t)={R}_{C}{C}_{f}^{i}(t)$$

After the reproductive stage, the accumulated resource becomes $\,{L}_{T}-{C}_{a}^{i}(t)={L}_{T}-{R}_{C}{C}_{f}^{i}(t)$.

The RBM is a one-dimensional map modelled by Eqs (10–12), where −*R*_*C*_ is the slope at the fixed point of the RBM. Isagi (1996) introduced the fruiting efficiency of a tree, *Y*(*t*)^16^, as a global coupling term.

13 $$Y(t)={[\frac{1}{(N-1){P}_{0}}\mathop{\sum }\limits_{j=1,j\ne i}^{N}{C}_{f}^{i}(t)]}^{\beta }$$

where *β* is the strength of pollen coupling and *N* denotes the population size.

Equation (12) is replaced by Eq. (14) to model the pollen coupling:

14 $${C}_{a}^{i}(t)={R}_{C}{C}_{f}^{i}(t)Y(t)$$

As described above, the GCM-RBM is established with Eqs (10–14).

To model the phase synchronisation associated with a nontrivial disturbance, we incorporate individual noise (*e*_I_) and common noise (*e*_C_) into the GCM-RBM. Individual noise (*e*_I_) assumes the heterogeneity of trees^24^. Common noise (*e*_C_) can induce synchrony, which is known as the Moran effect^25^.

These noise types are imposed on *P*_S_ in the following manner:

15 $${P}_{S}^{i}(t)={P}_{0}\{1+{e}_{{\rm{C}}}\sigma (t)\}\{1+{e}_{{\rm{I}}}{\delta }^{{\rm{i}}}(t)\}.$$

Here, the random number *σ*(*t*) is drawn from the normal random number *N* (*μ*, *σ*^2^) = (0, 1), and *δ*^i*i*^(*t*) is assigned to each tree individually. *P*_0_ is the intrinsic annual surplus. The seed production level *x*_*i*_(*t*) obtained in the survey (Fig. 1(a)) is considered proportional to ${C}_{S}^{i}(t)={C}_{f}^{i}(t)Y(t)$ in the GCM-RBM. In the numerical simulations, *P*_0_ and *L*_*T*_ were set as 10 and 100, respectively,

## Results and Discussion

### Field experiments

Figure 1 shows the results obtained in the primary survey at Fuchu City. The seed production level of the individual trees, *x*_*i*_(*t*) and annual (ensemble) mean of the population at year *t*, $X(t)=\frac{1}{N}\mathop{\sum }\limits_{i=1}^{N}{x}_{i}(t),$ are shown in Fig. 1(a). *X* (*t*) mostly shows period-3 cycles of an on-year (high-production year) followed by two consecutive off-years (low-production years). Figure 1(b) shows the histograms of seed production level. Here, the on-year of the population was determined by the median of $O{N}_{P,i}(t),\,i=1,2,\ldots ,N$ for *t* = 2003, 2005, 2008, 2011, 2014 and 2016. The histogram for the 15-year total is shown in the last subplot of Fig. 1(b) in red, showing that 70.3% (approximately two-thirds) of the counts are classified in the two lowest classes: 0–10and  11–20. In Fig. 1(c), the fraction of periods is represented by *FP*_*i*_(*Q*) and *FP*(*Q*) for each individual tree and the population, respectively. In *FP*_*i*_(*Q*) plots, several individual trees showed components of period-4 and/or larger periods; however, it is clearly identified that the period-3 is dominant followed by period-2 for these plots, i.e., *FP*(3) = 0.5354 and *FP*(2) = 0.1860. It is clear that the components of period-4 and larger periods are trivial, e.g., *FP*(4) = 0.0252, *FP*(5) = 0.0896 and *FP*(6) = 0.0236. Thus, *FP*(Q) defined in Eq. (7) is a powerful tool to quantify the components of periods in an ensemble time-series data set. The phase *θ*_*i*_(*t*) calculated by Eq. (1) for all trees is plotted in Fig. 1(d) and the median of {*θ*_*i*_(*t*)} was employed as the representative phase *Θ*(t) of the population, which illustrates the period-3 sequence [ON ⇒ OFF ⇒ OFF ⇒ ON] corresponding to [0 ⇒ 2π/3 ⇒ −2π/3 ⇒ 0] consisting of the three fundamental phases (0, 2π/3 and −2π/3). Figure 1(e) illustrates the annual changes of the phase histograms and the three fundamental phases are indicated by the three dashed lines. The fundamental phases (0, 2π/3 and −2π/3), correspond to the on-year (0), the first successive off-year (2π/3) and the second successive off-year (*−*2π/3), respectively. The population of *θ*_*i*_(*t*) is centred at one of the three fundamental phases. The last subplot of Fig. 1(e) in red, which shows the phase histogram for the 15-year term, clearly demonstrates the three evenly distributed peaks corresponding to the three fundamental phases (0, 2π/3 and −2π/3). Figure 1(f) displays a circle map in each panel in which blue dots are plotted at the points of (cos*θ*_*i*_(*t*), sin*θ*_*i*_(*t*)). On each panel, the arrow represents the vector (mean{cos*θ*_*i*_(*t*)}, mean{sin*θ*_*i*_(*t*)}), and the amplitude of the arrow is the order parameter that measures the strength of synchronisation. In the panel for 2003 in Fig. 1(f), the amplitude of the arrow is almost zero. This indicates that desynchronisation occurred in 2003. Therefore, this year should not be an ‘on-year’ even though *Θ*(2003) is almost zero. This is consistent with the median of {*ON*_*P,i*_(*t*), *i* = 1, 2, … *N*} indicating an ‘off-year’ in *t* = 2003. Hence, the phase *θ*(*t*) is useful to diagnose the states of the synchronisation of ensembles of trees. Thus, in Fig. 1, we detected a period-3 dominant phase synchronisation of *Zelkova serrata*. Period-2 synchronisation is common as alternate bearing in crop production, such as that of citrus^39–41^ and nuts^42^. In addition, in acorn masting, irregular and/or intermittent sequences are also common^1,43,44^. Therefore, the dominance of the period-3 sequence observed in *Zelkova serrata* is unique and notable. The masting of *Zelkova serrata* is characterised by two key features: (a) two significant periods, i.e., period-3 and period-2 coexist, and (b) the fraction of period-3, *FP*(3), is significantly larger than that of period-2, *FP*(2).

Figure 2 shows the results of the additional  survey at Shinjuku District. The key features observed in the primary survey at Fuchu City shown in Fig. 1 are completely consistent with those of the additional survey at Shinjuku District. In particular, the agreements identified in the histograms of seed production (Figs 1(b) vs 2(b)) and the histograms of phase (Figs 1(b) vs 2(b)) are remarkable. The results indicate the presence of a long-range spatial synchronisation between the two populations at Fuchu City and Shinjuku District. The ON–OFF sequence of the two populations was perfectly matched from 2006 to 2017. The presence of such strong spatial synchronisation suggests that global pollen coupling occurred in the range of at least 20 km. The long-ranged spatial synchronisation of masting also has attracted widespread interest^45^.

#### Mechanism of period-3

Figure 3 plots the density bifurcation diagrams of *S* and *θ*_Cs_ for three *β* values. For *β* = 0, in Fig. 3(a), the first bifurcation occurs at *R*_C_ = 1, and at the even integer values of *R*_C_, such as 2, 4, …, periodic solutions appear for *S*. For *β* = 6, in Fig. 3(b), the bifurcation diagrams of *S* display period-(*Q* + 1) windows in the interval where *R*_C_ = *Q* belongs^17^. The period of the periodic window increases as *R*_*C*_ increases. This bifurcation is known as the period-adding bifurcation, which is a typical property of border-collision bifurcation explained in Fig. 4^46,47^. The period-adding sequence is clearly demonstrated in Fig. 3(b,f). The density bifurcation diagrams of *θ*_Cs_ are also displayed in Fig. 3(c,d) for *β* = 0 and 6, respectively. It is difficult to measure *S* for a real tree but it is possible to measure *C*_*S*_. Therefore, the phase (*θ*_*Cs*_) is a powerful variable to investigate the periods of real-world masting data. The fractions of period-*Q* {*FP*(*Q*); *Q* = 2 …, 6} defined by Eq. (7) are expressed in Fig. 3(e,f) for *β* = 0 and 6, respectively. With pollen coupling of *β* = 6, *FP*(*Q*) is dominant (mostly 1.0) in the interval of the period-*Q* window, as shown in Fig. 3(f). This result indicates that the perfect period-(*R*_*C*_ + 1) phase synchronisation arises in the ranges including every digit number of *R*_*C*_^17^. In the ranges between adjoining period-windows, we can determine the composition of various period-*Q* (*Q* = 2, 3 …) with *FP*(*Q*). The strength of phase synchronisation is estimated as *F*_IN_. For example, in Fig. 3(e), at up *F*_IN_ is 0.5 or smaller because, there is no-coupling as *β* = 0. Contrarily, for *β* = 6, *F*_IN_ is maintained at 1 for the entire range of *R*_C_ shown in Fig. 3(f), which indicates perfect phase synchronisation.

For *β* = 6, a clear period-3 window is present in the range 1.6172 ≤ *R*_*C*_ ≤ 2.0 (Fig. 3(b)). Therefore, we selected *R*_*C*_ = 1.6171 to generate Fig. 4. The first iterated map is plotted in Fig. 4(a) with thick black dots for *β* = 6. The first iterated map for *β* = 0 is drawn in a thin blue line, which is a tent map as formulated by Eq. (10). The two first iterated maps of Fig. 4(a) are identical only in *S*(*t*) < *L*_T_. At the border, *S*(*t*) = *L*_*T*_, the left derivative is 1 for both *β* = 0 and 6. The right derivatives are −*R*_C_ and 0 for *β* = 0 and 6, respectively. The third iterated maps for *β* = 0 and 6 are plotted in Fig. 4(b). The map for *β* = 6 exhibits a clear tangent bifurcation with the three tangency points of 84.4523, 94.4532 and 99.9438. These three tangency points are expressed with three circle marks. Figure 4(c,d) magnify the ranges where the two tangency points of 99.9438 and 84.4523 locate, respectively. The period-3 window of the GCM-RBM is explained by a tangent bifurcation^48,49^. Given that the map (*β* = 6) illustrated in Fig. 4(a) is a piecewise smooth and piecewise monotonic map, the bifurcation is the border-collision bifurcation causing the period-adding bifurcation^29,30^ as observed in Fig. 3(b,f). This border-collision bifurcation has been studied in the dynamics of switching circuits^29,30^. The existence of the identically same dynamics in electronic circuits and perennial plants implies the universality of the boundary-collision bifurcation in nature.

#### Effects of noise on the GCM-RBM

The effect of the two types of noise (*e*_C_) and (*e*_I_) on the GCM-RBM was evaluated with four combinations of *e*_C_ and *e*_I_ at *R*_C_ = 2 and *β* = 6 in Fig. 5(a–d). In the noise-free condition (Fig. 5(a)), perfect period-3 phase synchronisation is apparent. In the case of only common noise, *e*_C_ = 0.2 (Fig. 5(b)), perfect phase synchronisation is maintained, but four or more consecutive off-year sequences (i.e., larger periodic, irregular and/or intermittent) appear. It should be noted that imposition of common noise (*e*_C_) does not generate any individual disturbances.

Figure 5(c) shows the case where only individual noise *e*_I_ is imposed. The period-3 sequences are clearly identified with some disturbance as observed in Figs 1(a), 2(a). Figure 5(d) demonstrates significant disturbances; however, the appearance of four or more consecutive off-year sequences is inconsistent with the key features of the survey data for *Zelkova serrata*. It is obvious that the individual variability *e*_I_ is essential, and the common noise *e*_C_ is unnecessary, to explain the period-3 dominant synchronisation of *Zelkova serrata*. Conversely, irregular, intermittent sequences and/or long consecutive off-years have been reported in many other tree species, such as spruce, beech and oak^50^. These reported features correspond to the common noise-imposed case exhibited in Fig. 5(d). Thus, the imposition of both *e*_I_ and *e*_C_ could account for various masting behaviours^51,52^.

In Fig. 6, the density bifurcation diagrams with individual noise *e*_I_ = 0.2 are plotted in the same arrangements as in Fig. 3. It is difficult to obtain clear structural information from *S* in Fig. 6(a,b) because of noise presence (*e*_I_ = 0.2). However, even with noise, the density bifurcation diagrams of *θ*_Cs_ show clear structural information (Fig. 6(c,d)) indicating the presence of three fundamental phases in the wide range of *R*_C_. By varying *R*_*C*_, three states are observed in Fig. 6(d,f). In state I, the majority of RBMs behave randomly such that *F*_IN_ is much smaller than 1.0. In state II, *F*_IN_ attains 1.0 and the period-2 cycle dominates. In state III, period-3 and larger periods emerge, and the fraction of period-2 *FP*(2) gradually declines. The densities around the three fundamental phases are significantly high in state III as shown in Fig. 6(d).

#### Parameter studies: estimation of *β* and *R*_*C*_ for the field data

To estimate the range of *R*_*C*_ and *β* for the survey data for *Zelkova serrata*, we conducted a parameter study and present the results in the *β*–*R*_*C*_ diagrams of Fig. 7. First, we show the noise-free conditions in Fig. 7(a–c) corresponding to the fractions of period-2 *FP*(2), period-3 *FP*(3) and period-4 *FP*(4), respectively. The region of *FP*(2) = 1 is sickle-shaped, and those of *FP*(3) = 1 and *FP*(4) = 1 are wedge shaped.

For a noise-imposed condition (*e*_I_ = 0.2), *FP*(2), *FP*(3) and *FP*(4) are plotted in Fig.7(d–f), respectively. *F*_IN_ and *FP*(3)/*FP*(2) are shown in Fig. 7(g,h). The values *FP*(3) = 0.5354 and *FP*(3)/*FP*(2) = 2.879 were calculated for the primary survey data in Fig. 1. In Fig. 7(e), the range of *FP*(3) = 0.5354 is located above the sickle-shaped region. *FP*(3)/*FP*(2) = 2.879 also appears in the sickle-shaped region (Fig. 7(h)) and indicates the coexistence of period-3 and period-2 to the same extent as in Fig. 1. The strength of synchronisation is quantified by *F*_IN_. *F*_IN_ is 1 in state II. The *β*–*R*_C_ diagram of *F*_IN_ is divided into three regions corresponding to the states I, II and III (Fig. 7(g)). State I is the desynchronised region. In state II, *FP*(2) and *F*_IN_ are near or equal to 1.0, thus indicating almost perfect period-2 synchronisation. State III has phase synchronisation consisting of several different periods. The perfect period-(*R*_C_ + 1) synchronisation is apparent in every digit number of *R*_C_^17^ owing to the period-adding nature of border-collision bifurcation. With increasing noise level *e*_I_, the perfect phase synchronisation moves into imperfect phase synchronisations accompanied with partial desynchronisation. Some fractions of period-(*R*_C_ + 1) are still located in the region around the digit numbers of *R*_C_ and coexist with other periods. For example, the relatively higher intensity region of *FP*(2), *FP*(3) and *FP*(4) locates around *R*_*C*_ = 1, 2 and 3, as shown in Fig. 7(d–f), respectively. As discussed above, we estimated that *R*_*C*_ = 2, *β* = 6 are *e*_I_ = 0.2 are appropriate values for *Zelkova serrata*.

**Figure 8:**
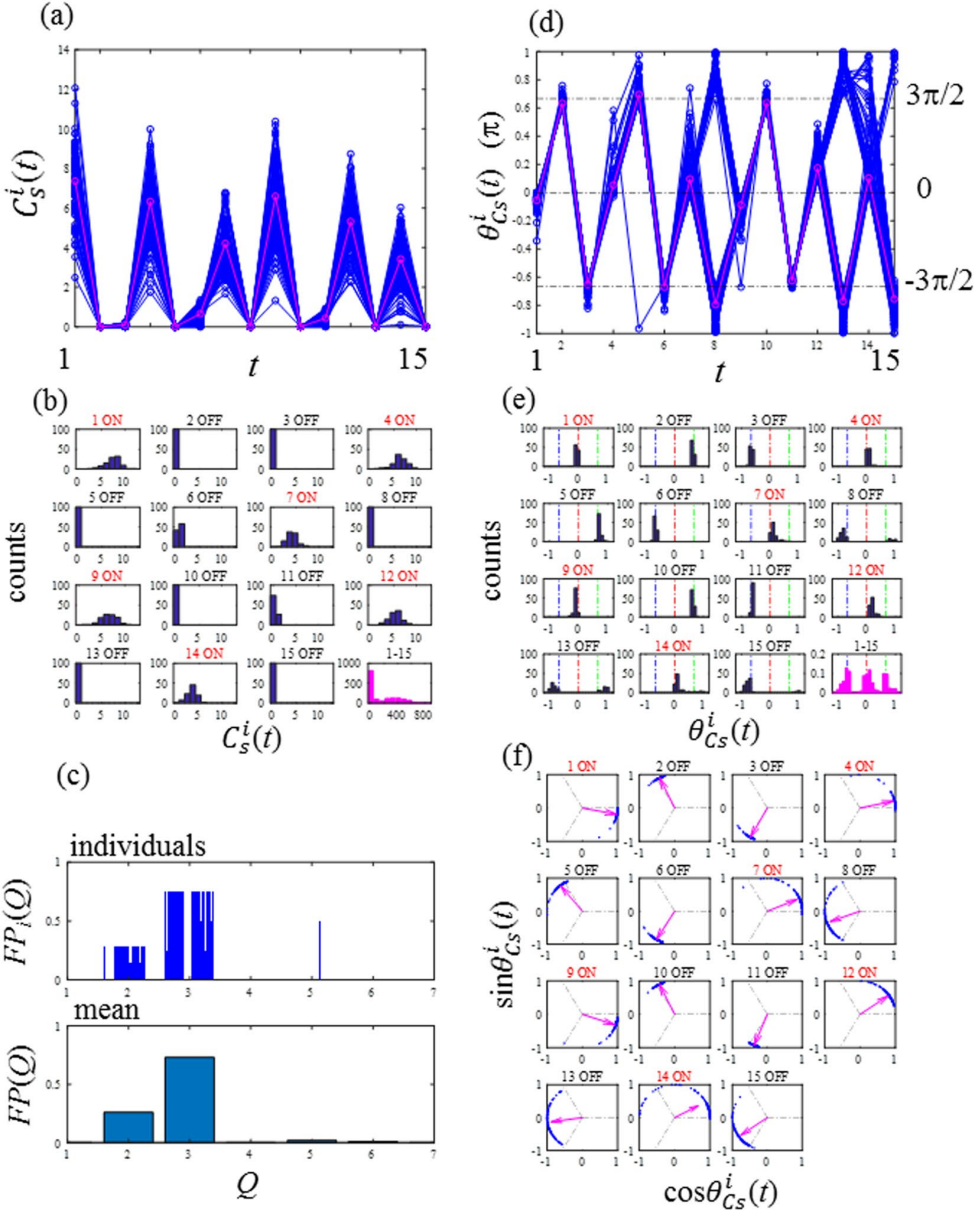
Period-3 phase synchronisation simulated by the globally coupled map of the resource budget model for *R*_*C*_ = 2, *β* = 6, *e*_I_ = 0.2 and *e*_C_ = 0. (**a**) Seed production level ${C}_{S}^{i}(t)$. The average production (thick solid line) demonstrates a clear period-3 pattern. (**b**) Histograms of ${C}_{S}^{i}(t)$. (**c**) Fractions of period-*Q* for _i_ndividuals, *FP*_*i*_(*Q*), and the population, *FP*(*Q*). (**d**) Phase of the seed production *θ*^*i*^_*Cs*_(*t*). (**e**) Histograms of *θ*^*i*^_*Cs*_(*t*). (**f)** Circle maps.

Figure 8 shows the results of a numerical experiment of the GCM-RBM with *R*_*C*_ = 2, *β* = 6, *e*_I_ = 0.2 and *e*_C_ = 0. The first key feature of period-3 synchronisation, which is an on-year followed by two consecutive off-years, is clearly demonstrated as most of the seed production (*C*_*S*_) behaves as a period-3 sequence (Fig. 8(a)). In Fig. 8(b), 70% of the counts were in the two lowest classes in the last panel. Fraction of periods, *FP*_*i*_(*Q*) and *FP*(*Q*), were demonstrated in Fig. 8(c) for individual trees and the population, respectively. The *FP*(*Q*) is determined as *FP*(2) = 0.260, *FP*(3) = 0.730, *FP*(4) = 0.020, *FP*(5) = 0.010 and *FP*(6) = 0.000. Period-3 is dominant followed by period-2 as *FP*(3)/*FP*(2) = 2.879, and the other periods are trivial. Three fundamental phases (0, 2π/3, and −2π/3) are repeated (Fig. 8(d,e)). In the last subplots of Fig. 8(e), the phase *θ*^*i*^_*Cs*_ is evenly located around the three fundamental phases. Importantly, the results presented in Fig. 8 are consistent with those of Figs 1 and 2.

### Concluding remarks

Synchronisation of seed production is ubiquitous in many tree species. In this paper, we clarified the mechanism underlying the period-3 dominant synchronisation in populations of *Zelkova serrata* surveyed in Tokyo. We developed a method to determine various periodic compositions coexisting in an ensemble time series. With this method, we found that the phase synchronisations of the populations of *Zelkova serrata* in Fuchu City and Shinjuku District are identical. The observed long-range spatial synchronisation implies global pollen coupling occurred in the range of at least 20 km.

We employed the GCM-RBM to describe the synchrony of the masting of *Zelkova serrata*. When the coupling of the GCM-RBM is sufficiently strong, the dynamics of the GCM-RBM become a piecewise smooth and piecewise monotonic map that is characterised by border-collision bifurcation and the period-adding bifurcation. The mechanism generating period-3 is explained as the tangential bifurcation of the GCM-RBM. The GCM-RBM realised the period-3 dominant phase synchronisation and the key features of the survey data.

The presence of a period-3 solution implies the coexistence of various periodic solutions and chaos in the system. In addition, the GCM-RBM generates periodic solutions larger than period-2 because of its period-adding bifurcation mechanism. Thus, the developed GCM-RBM shows the potential to describe diverse seed-production behaviours observed in many tree species by manipulating its control parameters (*R*_*C*_ and *β*) and the levels of individual and common noise imposed (*e*_I_ and *e*_C_).

## Supplementary information

Sample photo of Zelakova serrata in the primary survey
