# Supplementary material for: Period-3 dominant phase synchronisation of Zelkova serrata: border-collision bifurcation observed in a plant population
Source: Sci Rep. 2019 Oct 30;9:15568. doi: 10.1038/s41598-019-50815-8 (PMC6821922; doi:10.1038/s41598-019-50815-8)
Supplement: Supplementary file 1 — Sample photo of Zelakova serrata in the primary survey [file 41598_2019_50815_MOESM1_ESM.docx]

Supplementary information

**Period-3 dominant phase synchronisation of *Zelkova serrata*: border-collision bifurcation observed in a plant population**

**Kenshi Sakai^1*^, Yoshinobu Hoshino^2^, Awadhesh Prasad^3^, Atsuko Fukamachi^2^, Akira Ishibashi^2^**

^1^Department of Environmental and Agricultural Engineering, Faculty of Agriculture, Tokyo University of Agriculture and Technology, Tokyo 183-85-9, Japan

^2^ Department of Environment Conservation, Faculty of Agriculture, Tokyo University of Agriculture and Technology, Tokyo 183-85-9, Japan

^3^ Department of Physics and Astrophysics, University of Delhi, Delhi 110007, India

*Corresponding author. Tel: +81-42-367-5755. E-mail: ken@cc.tuat.ac.jp

Sample photo to show the difference of non-fruiting twig leaves area and fruiting leaves area in the primary survey

The leaf colour changes to vivid red in autumn and the leaves on fruiting twigs change colour much earlier than those on non-fruiting twigs.

Supplementary Figure. S1 Sample photo of *Zelakova serrata* in the primary survey

(a) *Zelakova serrata* tree no. 10 photographed from the roof top of Building no. 19 on Fuchu Campus, Tokyo University of Agriculture and Technology (18 October, 2018). Leaves on fruiting twigs are red, whereas those on non-fruiting twigs are still green. (b) Fruiting twig with seeds. This photo was taken by Akira Ishibashi.
